# A case report of necrotizing soft tissue infection of the chest wall: Effective management with serial debridement

**DOI:** 10.1016/j.ijscr.2021.105908

**Published:** 2021-04-27

**Authors:** Masakazu Fujii, Kiyotaka Imamura, Kentaro Kato, Minoru Takada, Yoshiyasu Ambo, Fumitaka Nakamura, Satoshi Hirano

**Affiliations:** a Department of Surgery, Teine Keijinkai Hospital, Sapporo, Hokkaido, Japan; b Department of Gastroenterological Surgery II, Hokkaido University Faculty of Medicine, Sapporo, Hokkaido, Japan

**Keywords:** Necrotizing soft tissue infection, Chest wall, Serial debridement, Group A streptococcus

## Abstract

**Introduction:**

Necrotizing soft tissue infection (NSTI) of the chest wall is a rare, rapidly spreading, highly lethal surgical disease. Radical debridement interferes with the important anatomical function of the chest wall. We report a case of chest wall NSTI that was successfully managed with early diagnosis and serial debridement.

**Presentation of case:**

A 43-year-old, previously healthy woman presented with severe malaise and worsening right axillary pain. She was severely lethargic and had a painful, large, pale lesion with surrounding erythema of the right chest and trunk. Computed tomography revealed NSTI, with diffuse soft tissue inflammation extending from the axilla to the lower abdomen. There was no obvious entry portal. Prompt surgical drainage was established. Group A streptococcus infection was diagnosed. During her 3-month postoperative course, she underwent four more surgeries, including two debridements. This treatment proved successful and avoided the need for complicated muscle flap reconstruction. She was discharged on postoperative day 109.

**Discussion:**

Group A streptococcus can cause NSTI even in immunocompetent patients without an entry portal. Radical debridement is recommended for infection control. Preserving anatomical chest wall function, however, is also important. Serial debridement with close follow-up solved the problem in this patient.

**Conclusions:**

Serial debridement with close follow-up enabled to avoid large tissue deficits and complicated reconstruction in the case of NSTI of the chest wall.

## Highlights

• Necrotizing soft tissue infection of the chest wall is rare and lethal.
• Radical debridement could interfere with the anatomical function of the chest wall.
• Adequate drainage with minimum skin incision avoids large tissue defects.
• Serial debridement with close follow-up enabled to avoid complicated reconstruction

## 1 Introduction

Necrotizing soft tissue infection (NSTI)—a surgical emergency associated with significant morbidity and mortality—is evidenced by necrosis and microvascular thrombosis in the subcutaneous fat, fascia, and/or muscle [1]. A review article of 20 cases of chest wall NSTI reported a mortality rate of 60%, with 15 patients having predisposing conditions [2]. We describe a case of chest wall NSTI due to group A streptococcus in an immunocompetent patient with no underlying medical disease. Because of the early diagnosis and serial debridement with closed follow-up, the patient survived without requiring complicated chest wall reconstruction. This case is reported in line with SCARE criteria [3].

## 2 Case presentation

**Fig. 1:**
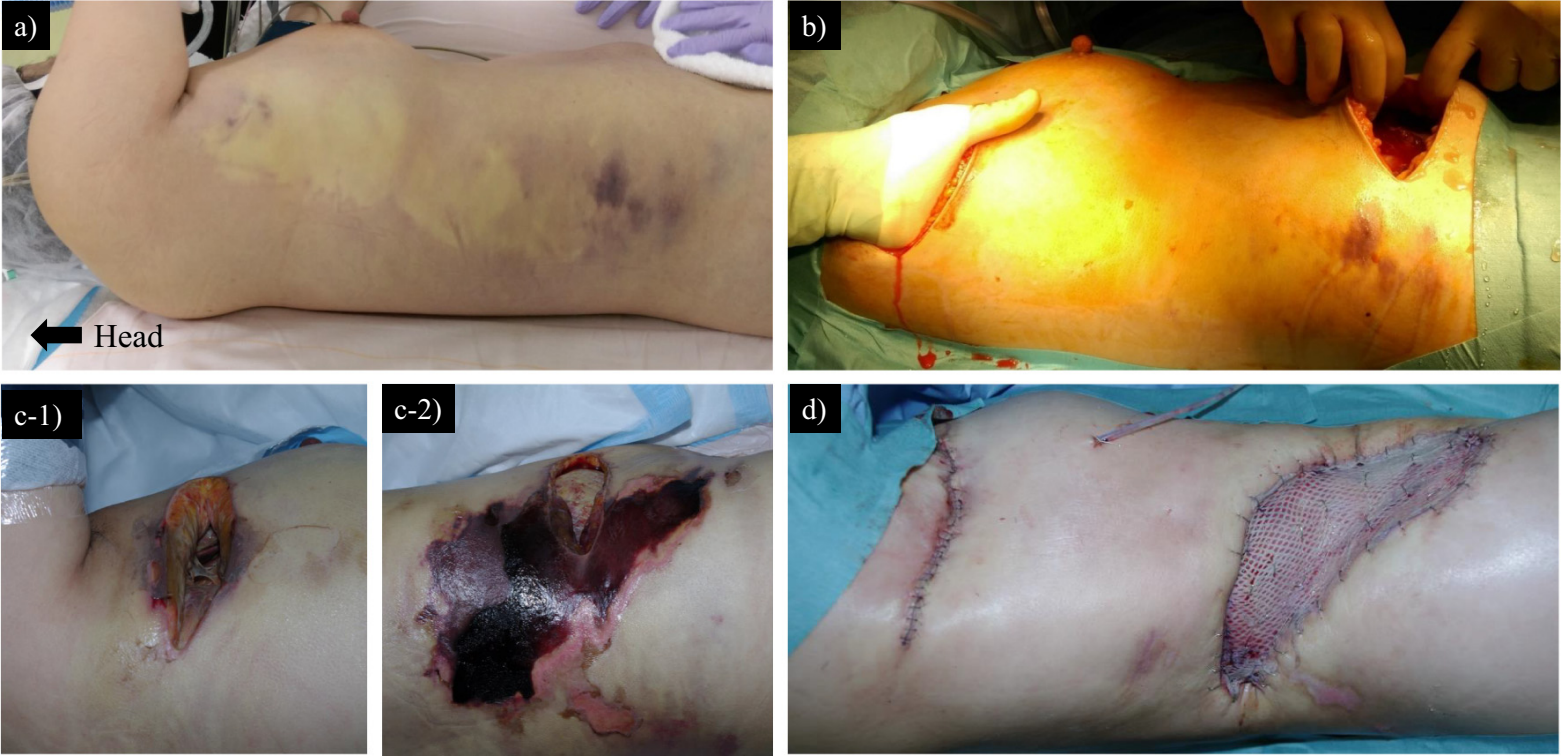
**a** Skin findings before the first surgery. A large pale lesion was found in the right chest and trunk, with hemorrhagic changes in the surrounding skin. **b** Horizontal incisions in the chest and abdomen at the first surgery. **c** Skin findings around the initial incisions of the chest (**c-1**) and abdomen (**c-**2) on postoperative day 11. **d** Wound closure on postoperative day 88. Abdominal wound was closed with a split-thickness skin graft from the right thigh.

**Fig. 2:**
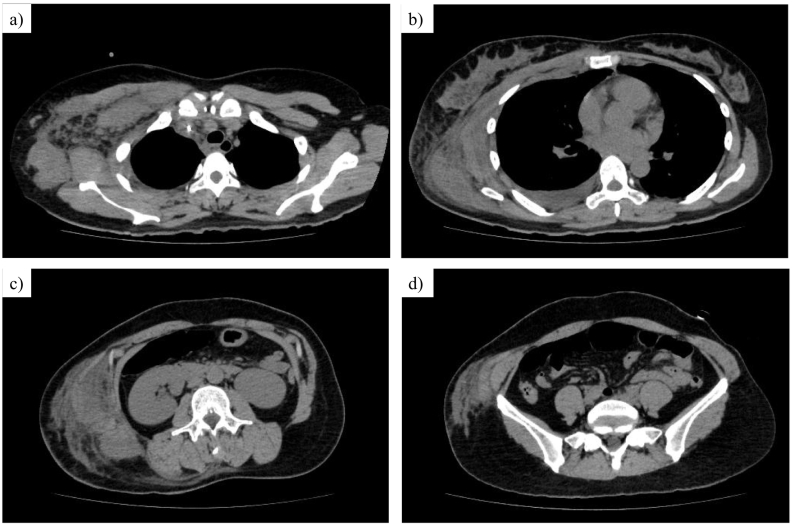
Computed tomography reveals diffuse soft tissue inflammation from the right axilla extending to the ipsilateral lower abdomen. It involved multiple lymphadenopathic areas in the axilla (**a**), a swollen mammary gland (**b**), and severe inflammation between oblique muscles (**c, d**).

A previously healthy 43-year-old Japanese woman presented with a 3-day history of fever and worsening right axillary pain. On admission, the pain was accompanied by severe malaise that rapidly progressed. She denied trauma or recent surgery. Her past medical history was insignificant (cesarean section >10 years previously). She was afebrile but tachypneic. Physical examination showed a large, pale lesion with surrounding erythema on the right chest and trunk (Fig. 1a). She was experiencing a disproportionate amount of pain, extending beyond the borders of the apparent skin findings. Laboratory tests showed the following: white blood cells 39,000/μL, hemoglobin 15.4 g/dL, creatinine 3.5 mg/dL, C-reactive protein 39 mg/dL, sodium (Na) 133 mmol/L, glucose 314 mg/dL, lactic acid 90 mg/dL. Computed tomography revealed diffuse soft tissue inflammation extending from the right axilla to the ipsilateral lower abdomen, along with lymphadenopathic areas in the axilla and a slightly swollen mammary gland (Fig. 2). The Laboratory Risk Indicator for Necrotizing Fasciitis score [4] was 11 (indicating high risk). Chest wall NSTI was highly suspected, and emergent surgical debridement was performed 3 h after presentation.

The patient was placed in left decubitus position for the surgery. Two 10-cm transverse incisions were made. The cephalad line was at the height of the nipples to accommodate drainage between the pectoralis and latissimus dorsi muscles. The caudal line, at the height of the umbilicus and lateral to the rectus muscle, was used for drainage between the external and internal oblique muscles and the inguinal region (Fig. 1b). Blunt dissection showed a lack of resistance of normally adherent fascia, and copious grayish exudate. The two dissection planes were connected, sparing the skin and subcutaneous fat layer. After washing the wound thoroughly, nine drains were placed in the dissected space for future drainage. The next day, we performed a second-look operation. We removed nonviable tissue but preserved the gray area for later evaluation regarding the appropriate extent of further debridement.

**Fig. 3:**
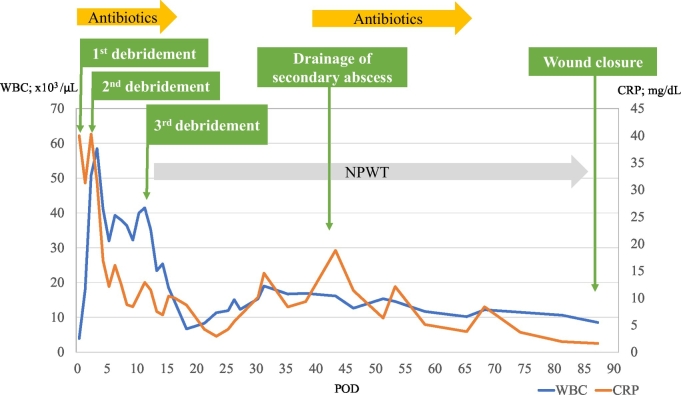
Clinical course of the patient. WBC, white blood cell count; CRP, C-reactive protein; POD, postoperative day; NPWT, negative-pressure wound therapy.

Vancomycin, piperacillin/tazobactam, and clindamycin were empirically initiated. After wound culture results confirmed *Streptococcus pyogenes*, these antibiotics were replaced with penicillin G and clindamycin. Antibiotics were administered until POD 18. On POD 11, additional debridement was deemed necessary because further necrosis was found in preserved skin around the abdominal wound, (Fig. 1c). Negative-pressure wound therapy was started on POD 17. The wound was then irrigated every other day with copious amounts of saline. On POD 44, a secondary abscess in the latissimus dorsi caused by *Escherichia coli* was discovered and debrided. On POD 88, the patient's wound was successfully closed with a split-thickness skin graft obtained from the right thigh (Fig. 1d). On POD 109, she was discharged fully ambulant (Fig. 3).

## 3 Discussion

NSTIs can involve atypical anatomical sites, such as the chest wall. A published review of NSTI noted that, among those on the chest wall, most appear postoperatively [2]. In our patient, we considered lymphadenitis and mastitis as possible sources of the infection on the basis of previous reports [5,6], but we found no relevant history. Spontaneous cases without a portal of entry reportedly comprise nearly half of all NSTI cases caused by GAS [1]. Moreover, GAS can cause severe infections such as NSTI in populations with no underlying illness [7], and 15% are in the chest wall [8]. Our immunocompetent patient developed an NSTI with no entry portal. Although an unremarkable history and atypical patient background could lead to misdiagnoses, we should remain highly suspicious that an NSTI may be present.

In our case, the NSTI diagnosis was based on her rapidly progressive clinical course with pain disproportionate to the skin color changes. This unique physical finding has been reported as one of the red flags of NSTI [9]. Diffuse soft tissue inflammation, including fascial edema on computed tomography, confirmed the diagnosis. Clinical scoring systems such as the Laboratory Risk Indicator for Necrotizing Fasciitis score have been described as useful [4]. In our patient it was sufficiently high to suggest NSTI.

The amount of initial debridement in NSTI patients is controversial. Although radical resection has been recommended in terms of infection control [10], a large skin and soft tissue deficit would cause loss of anatomical function and make later reconstruction difficult. Another report described successful debridement of a chest wall NSTI using a long craniocaudal incision [11]. This large defect was later covered with a latissimus dorsi free flap. The chest wall has essential anatomical functions associated with respiration, thoracic organ protection, and upper limb motion. A full-thickness defect would require complicated reconstruction with muscle flaps [12]. Optimizing the extent of debridement is therefore paramount when taking into consideration functionality of the remaining chest wall and infection control. The reason for placing the two horizontal incisions for drainage during the initial operation was to minimize neurovascular bundle injuries, such as harm to the intercostal arteries and cutaneous branches of thoracoabdominal nerves.

Serial surgical debridement coupled with negative-pressure wound therapy (NPWT) has been reported as safe, and it saves skin and soft tissue if needed for flaps to treat Fournier's gangrene [13]. At her first presentation, our patient displayed extensive skin changes suggesting ischemia. We considered, however, that almost all the skin lesions were only partially necrotized and could be preserved. Therefore, we performed only drainage above the fascia without massive resection of the tissue. The first drainage attempt took place where manual dissection was available because the infection had weakened inter-layer adhesion. Although the preserved soft tissue around the abdominal incision later required resection, the tissue around the thoracic incision remained intact. When the infection was mostly controlled, we initiated NPWT with constant irrigation. The delayed appearance of an abscess in the back was thought to have been caused by insufficient drainage. The back is anatomically distant from the NPWT-attached site, and drainage fluid collection is easy. Skin- and soft tissue-sparing serial drainage, however, should be accompanied by careful follow-up of the remnant source of the infection.

## 4 Conclusions

NSTI should be highly suspected even at atypical sites in patients without predisposing conditions. Serial and optimized-range debridement may prolong the postoperative course but enabled to minimize skin and soft tissue deficits and to avoid complicated reconstruction.

## Declaration of competing interest

All authors have no conflict of interest about this study.
